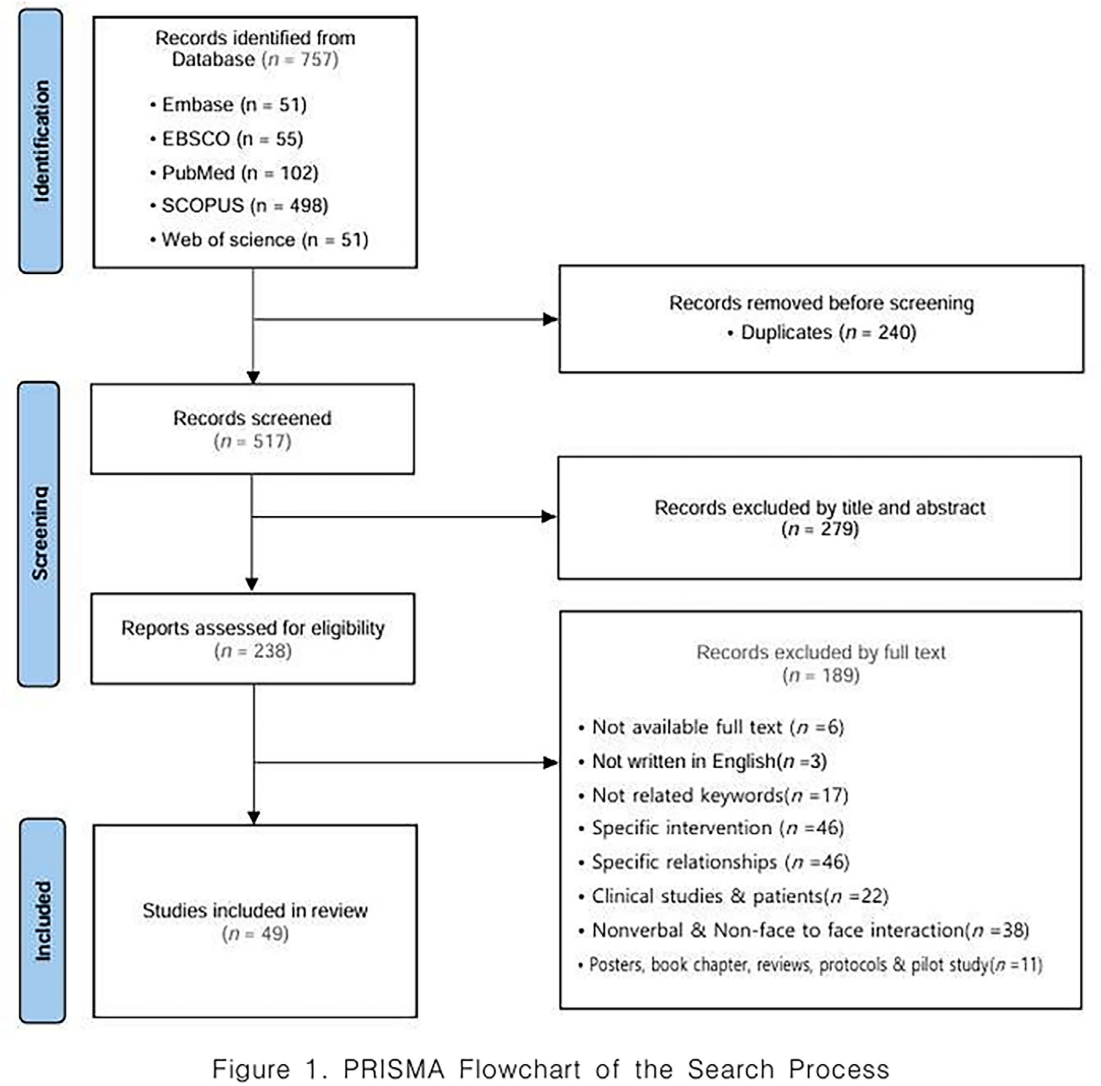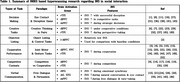# Integrated Understanding of Interpersonal Neural Synchronization (INS) in Social Interaction: A Systematic Review of fNIRS‐based Hyperscanning Studies

**DOI:** 10.1002/alz70861_108583

**Published:** 2025-12-23

**Authors:** Seyeon IN, Ji‐Hyuk Park

**Affiliations:** ^1^ Yonsei university, Wonju‐si, Gangwon‐do Korea, Republic of (South); ^2^ Yonsei University, Wonju, Gangwon‐do Korea, Republic of (South)

## Abstract

**Background:**

Social interaction is a fundamental aspect of human cognition, influencing daily functioning and social success. Recent advances in neuroimaging have enabled researchers to investigate the neural underpinnings of social interaction, particularly through the lens of interpersonal neural synchronization (INS). INS refers to the temporal alignment of neural activity between individuals engaged in social interaction, and it has emerged as a core concept in second‐person neuroscience. The development of hyperscanning techniques, especially those using functional near‐infrared spectroscopy (fNIRS), has allowed simultaneous brain activity recording from multiple individuals. Among these, fNIRS is noted for its non‐invasiveness, portability, and tolerance to movement, making it ideal for studying real‐world social interactions. Despite the growing body of research, systematic integration of findings across fNIRS‐based INS studies remains limited, calling for a structured review.

**Method:**

This systematic review was conducted based on the hypothesis that interpersonal neural synchronization (INS) serves as a neural marker for successful social interaction. A literature search was performed using five major international databases—Embase, EBSCO, PubMed, Scopus, and Web of Science (WoS). Publications from January 2020 to February 2025 were included. The search terms used were “fNIRS”, “hyperscanning”, “interpersonal neural synchronization”, “interpersonal brain synchronization”, “brain‐to‐brain coupling” and “interactive brain activity”

**Result:**

A total of 49 studies were included. INS predominantly occurred in the prefrontal cortex (PFC), dorsolateral prefrontal cortex (DLPFC) and temporoparietal junction (TPJ) across six main task types: decision‐making, creativity, objective listing, cooperative performance, competitive contexts, and verbal communication. INS consistently correlated with effective collaboration, emotional alignment, and strategic interactions, reinforcing its potential as a biomarker for successful social engagement. However, inconsistencies in interpretative frameworks and experimental designs across studies remain a significant limitation.

**Conclusion:**

This study confirms that INS serves as a meaningful neural marker in social interaction, providing significant evidence for understanding the social brain in humans. Furthermore, it opens new possibilities for quantifying and interpreting human social cognitive processes, suggesting the potential for practical applications in future social neuroscience research and various applied fields.